# Supplementary figures and images for: Non-specific protection from respiratory tract infections in cattle generated by intranasal administration of an innate immune stimulant
Source: PLoS One. 2020 Jun 25;15(6):e0235422. doi: 10.1371/journal.pone.0235422 (PMC7316291; doi:10.1371/journal.pone.0235422)

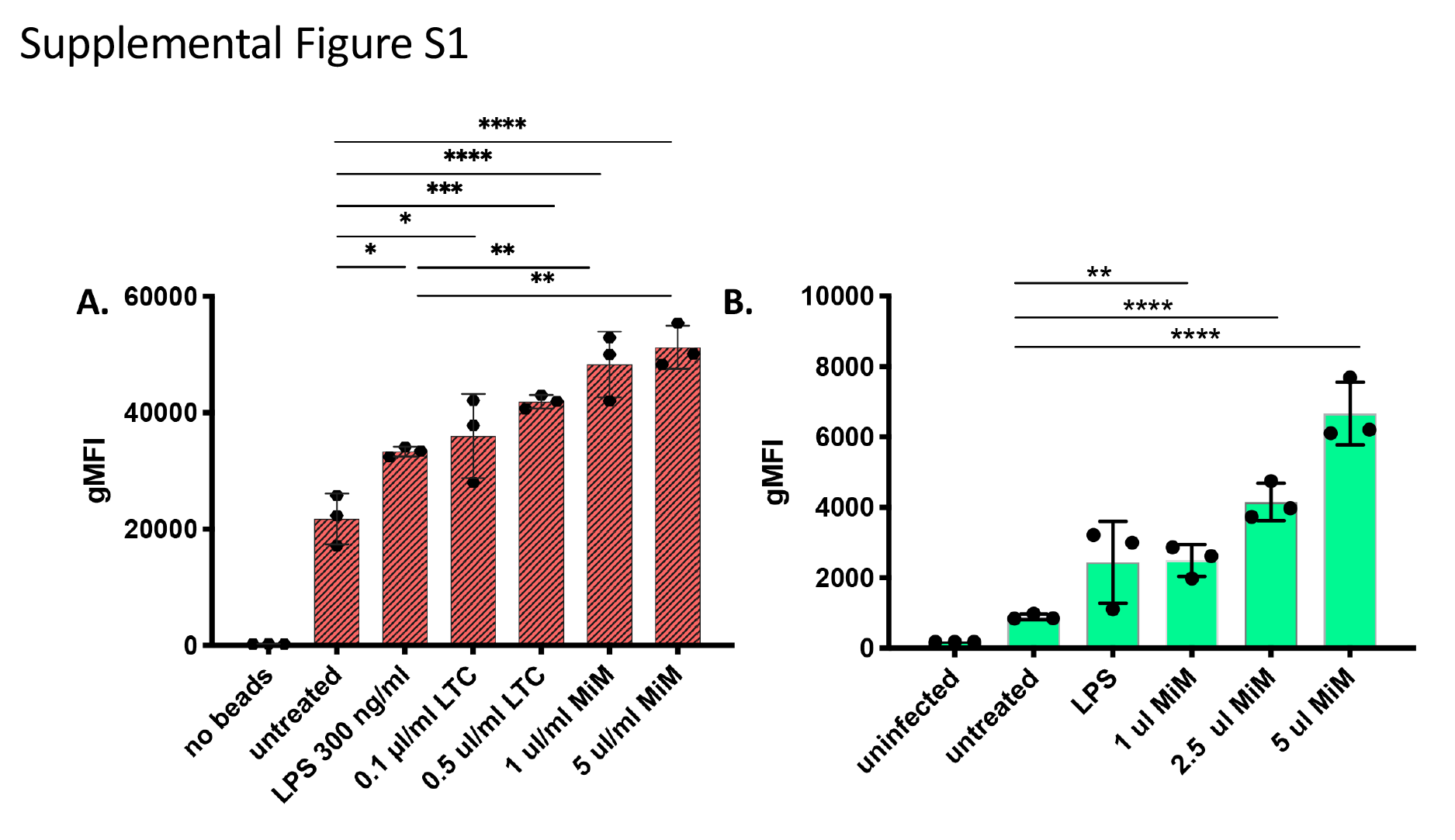

Supplement: S1 Fig — Triplicate cultures of MDM were either untreated, treated with LPS or with increasing concentrations of LTC for 24 h, followed by addition of fluorescent 1 um beads, as described in Methods (A). Cells were incubated with beads for 2 h and harvested by trypsinization followed by flow cytometric analysis to quantitate bead positive cells. In separate studies, MDM were incubated with S. aureus, and intracellular uptake quantitated by immunostaining for S. aureus, followed by analysis by flow cytometry. Macrophages were pre-treated with vehicle, LPS or increasing concentrations of LTC followed by infection with S. aureus. Analysis of variance of gMFI parameters was evaluated using a two-way ANOVA with, *, P<0.05; **, P <0.01; ***,P<0.005 and ****, P<0.0001. (TIF) [file pone.0235422.s001.tif]

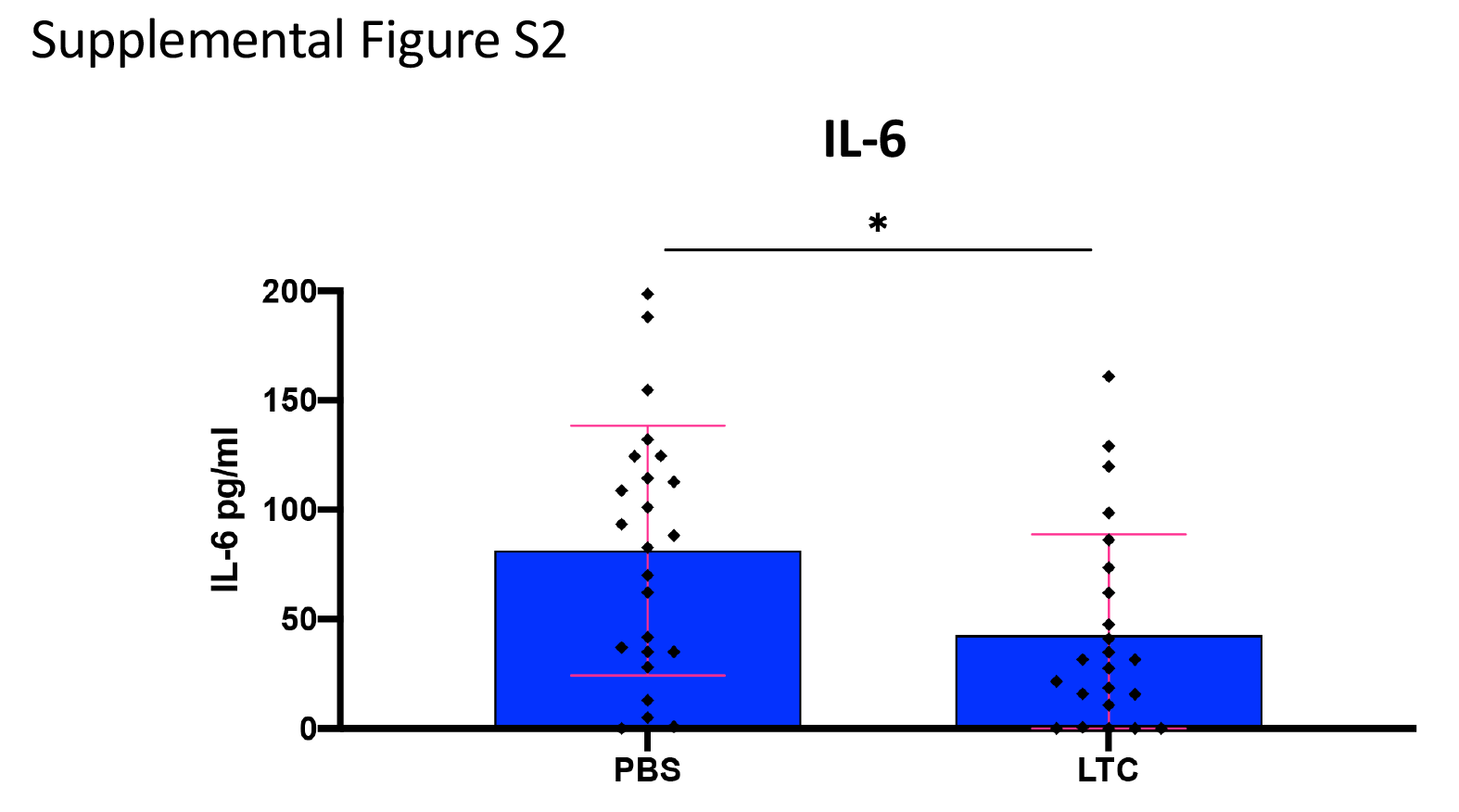

Supplement: S2 Fig — Cattle were treated with PBS or LTC administered by the intranasal route (2 ml per nostril) prior to exposure to BRDC seeder animals. Bronchial swabs were obtained at necropsy in animals that were euthanized due to BRDC or study completion (24 days). Bronchial samples were analyzed for IL-6 concentrations by ELISA. Statistical comparisons of differences in cytokine release was analyzed by ordinary one-way ANOVA with multiple comparisons with, *, P< 0.05. (TIF) [file pone.0235422.s002.tif]

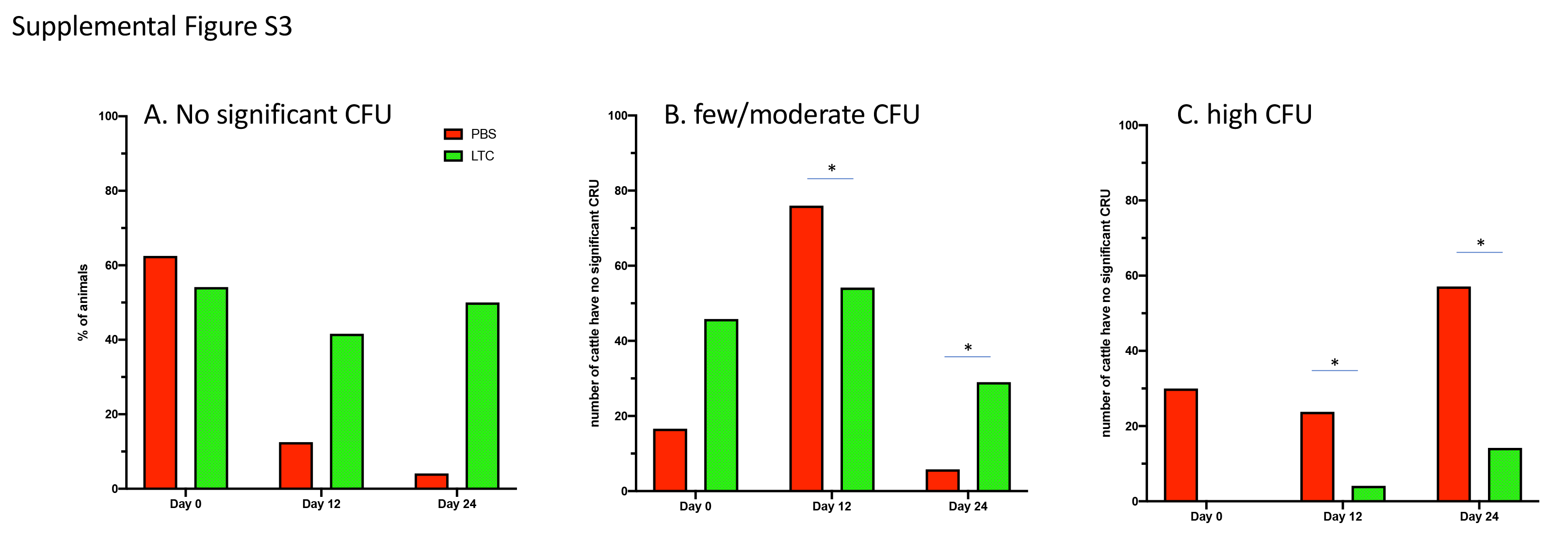

Supplement: S3 Fig — Nasal swabs were obtained from cattle and plated on Brain Heart Infusion or blood agar plates and CFU were analyzed and assessed for pathogenic strains of respiratory bacteria including Mannheima spp., P. multocida and Truperella pyogenes. Extent of infection was assessed as either “no significant CFU”(A), “few/moderate CFU (B) or “high CFU” (C). For day 24, CFU were obtained from lung necropsies. Data were analyzed for significance using an unpaired t test with *, P≤0.01. (TIF) [file pone.0235422.s003.tif]
